# On Some Recent and Remarkable Examples of the Protection Afforded by Metallic Conductors against Heavy Strokes of Lightning

**Published:** 1847-12

**Authors:** W. S. Harris


*On some Recent and Remarkable Examples of the Protection Afforded by Metallic Conductors against Heavy Strokes of Lightning.* By Sir W. S. Harris.—The possibility of guarding buildings and other structures against the destructive effects of lightning has been made a great question in practical science, from the time of Franklin to the present day; and it is of considerable public importance, seeing the frequent damage which occurs to our beautiful churches and other edifices by strokes of lightning, to bring this question completely under the dominion of induction, observation and experiment. The general principles which Sir W. S. Harris submitted as deducible from the inquiries to which he alluded are these: If we imagine a ship or building to consist altogether of metallic substances, it would certainly be secure from any damage by lightning, and for this simple reason, that what we call lightning is the result of the electrical agency forcing a path through resisting matter, such as the air, and extricating, with explosive and expansive force, both light and heat in its course. When, on the contrary, it falls upon comparatively nonresisting bodies, such as the metals, then this form of lightning vanishes, and the discharge assumes, if the metallic body be sufficiently capacious, the form of a comparatively quiescent current. Our object should be, therefore, in defending any building or ship from lightning, to bring the general mass so far as possible into that passive or comparatively nonresisting state it would have, supposing it a mass of metal. This is, in fact, the single and simple condition of such an application, without any reference whatever to assumed forces of attraction or peculiar specific powers manifested by certain bodies for the matter of lightning, and which really do not exist. This simple principle, by a careful mechanical arrangement calculated to render it practical and applicable to all the duties which the general structure of a ship, together with its masts, has to perform, is now universally carried out in the navy with the most perfect success; so that damage by lightning in the vessels so fitted has for the last fifteen years quite ceased. The masts are made completely conducting by capacious plates of copper reaching from the highest points to the keel, and are tied into one general connection with all the great metallic masses employed in the construction of the hull, and united, by the large bolts of copper passing through the keel and sides, with the copper expanded over the bottom and with the sea. It is quite impossible that a discharge of lightning can fall on the vessel in any place, and not be at once transmitted safely by the conductors, not under the form of lightning, but under the form of a current without explosion. Sir W. S. Harris then referred to some remarkable cases.—*Transactions of the British Association.—Jour. Franklin Institute.*

